# Mental health of South Korean adolescents in the COVID-19 Era: Web-based survey focused on suicide-related factors

**DOI:** 10.1186/s13034-023-00658-z

**Published:** 2023-10-13

**Authors:** Sang Mi Kim, Yeong Mi Jeong, Hye Seon Park, Sulki Choi

**Affiliations:** 1 Department of Health Management, Jeonju University, 303 Cheonjam-ro, Wansan-gu, Jeonju-si, 55069 South Korea; 2 Department of Health & Medical Administration, Gyeongnam Geochang University, Geochang, Gyeongnam South Korea; 3 College of Applied Health Science, Biomedical Health Information Science, University of Illinois at Chicago, 1919 W Taylor St, Chicago, IL 60612 USA

**Keywords:** Academic achievement, Adolescents, COVID-19, Inadequate sleep, Drug use, Mental health, Republic of Korea, Suicidal ideation, Suicide attempt, Suicide prevention

## Abstract

**Background:**

The coronavirus disease 2019 (COVID-19) pandemic has negatively impacted many aspects of life. Measures for preventing the spread of COVID-19 (e.g., school lockdowns, remote and hybrid classes, group and outdoor activity restrictions, and social distancing in the classroom and meal time) could have led to adolescents to experience anxiety and depressive symptoms. Such mental health impacts could increase the risk of suicidal ideation in this population. Moreover, according to a report by the Organization for Economic Co-operation and Development, although the total number of suicide deaths in South Korea decreased in 2021, the suicide rate of those aged 10–29 years increased. One factor affecting the result is adolescent mental health by COVID-19. This study examines the mental health status of South Korean adolescents amid the prolonged COVID-19 pandemic, and identifies and analyzes predictors of suicidal ideation, suicide planning, and suicide attempts.

**Methods:**

The study used data from 54,948 adolescents who participated in the 2020 Korea Youth Risk Behavior Web-based Survey. Based on their responses to suicide-related questions, the sample was divided into a healthy group, suicide-ideation group, suicide-planning group, and suicide-attempt group. The descriptive statistics of these groups were then analyzed. An analysis of covariance, post-hoc tests, and multiple logistic regression analysis were performed on the four groups.

**Results:**

Overall, 6.9% of the participants reported suicidal ideation, 2.2% reported planning suicide, and 1.9% reported attempting suicide in the previous 12 months.

**Conclusions:**

During the prolonged COVID-19 pandemic, there is a strong need for various individualized programs that identify and intervene to support adolescents at risk of suicide by accurately assessing their mental health risk factors, such as stress, sadness and despair, loneliness, and generalized anxiety disorder. Accordingly, it is necessary to develop and distribute a mental health and well-being curriculum, strengthen suicide prevention programs and support services, expand mental health diagnostic tests, and school-based mental health programs.

## Background

As a result of its high infectivity, the coronavirus disease 2019 (COVID-19) has spread rapidly worldwide and has caused many deaths. However, its impact is not limited to physical morbidity and mortality, as it has triggered a global financial crisis, substantial sociocultural changes, and modified individuals’ daily lives. Meanwhile, the 2003 Severe Acute Respiratory Syndrome (SARS) epidemic affected individuals across 30 different countries. A study that evaluated the mental health of a sample of SARS survivors from Hong Kong reported that 30 months after the epidemic had ended, 47.8% were still experiencing posttraumatic stress disorder and 25.6% were showing persistent mental health problems [1].

As a result of its high infectivity, the coronavirus disease 2019 (COVID-19) has spread rapidly worldwide and has caused many deaths. However, its impact is not limited to physical morbidity and mortality, as it has triggered a global financial crisis, substantial sociocultural changes, and modified individuals’ daily lives. Meanwhile, the 2003 Severe Acute Respiratory Syndrome (SARS) epidemic affected individuals across 30 different countries. A study that evaluated the mental health of a sample of SARS survivors from Hong Kong reported that 30 months after the epidemic had ended, 47.8% were still experiencing posttraumatic stress disorder and 25.6% were showing persistent mental health problems [1].

In May 2015, an outbreak of the Middle East Respiratory Syndrome reached South Korea, resulting in the closure of some schools and hospitals, and individuals with suspected symptoms were required to self-isolate for two weeks. A study of individuals who completed their isolation by mid-June 2015 found that by September to November 2015, 50% were still feeling anxiety and 50% were feeling anger; further, at approximately 4–6 months after their isolation, 20% continued to feel anxiety and 30% felt anger [2]. There is also evidence from various countries—China included—of the mental health problems evoked by COVID-19 on the general population, showing relatively high rates in the studied nations for the following problems: anxiety (6.33–50.9%), depression (14.6–48.3%), post-traumatic stress disorder (7–53.8%), psychological stress (34.43–38%), and stress (8.1–81.9%) [3].

In 2019, a community health survey report conducted in South Korea showed that 3.79% of the general population were in the depression risk group and 4.7% of the adults reported experiencing suicidal thoughts. Then, in September 2020, an online survey was conducted and showed that the percentage of people in the depression risk group had risen to 22.1% and the suicide thoughts rate was 13.8%. Moreover, results from the 2021 COVID-19 National Mental Health Survey, conducted by the Korean Trauma Stress Association and the Ministry of Health and Welfare, showed that the percentage of people in the depression risk group was 22.8% in March (decreasing to 18.9% in December), and that of people who reported experiencing suicidal thoughts was 16.3% in March (decreasing to 13.6% in December) [4]. The figures above show that depression rates and suicide thoughts decreased over time in South Korea [5]. There was another South Korean survey-based study that conducted, from April to August 2020, an investigation on the impact of the prolonged COVID-19 pandemic on the mental health of 500 adults; the percentages of people in the depression risk group in April and August were 18.4% and 23.2%, respectively, and those of people in the anxiety risk group were 11.8% and 16.0%, respectively [6]. Other studies remark that the psychological responses that emerge at the population level during an infectious disease outbreak can both accelerate the spread of the disease and trigger emotional distresses and social impairments that persist even after disease eradication [7, 8].

The first report of a COVID-19 patient in South Korea took place on 20 January 2020. Afterwards, in a single month, there was around 8,000 cumulative confirmed cases in Daegu and Gyeongsangbuk-do, making this the second largest number of cumulative confirmed cases, losing only to China. On 12 March 2020 (Korea Standard Time), the World Health Organization officially declared the COVID-19 pandemic. In August 15, after the Liberation Day rally, there was even a second wave of COVID-19 in two locations in Seoul, namely Gwanghwamun and the Sarang First Church in Seongbuk-gu. The government also imposed social distancing policies during the beginning of the pandemic, which led to the postponing of the opening of elementary, middle, and high schools three times. Accordingly, remote classes began on 9 April 2020, and in the second semester of 2020, real-time video classes became the common trend at all stages of education.

Adolescence is a key period for the development of one’s character; however, the continued threat of the COVID-19 pandemic means that many adolescents have been forced to spend some of their formative years in relative isolation. The consequent psychological anxiety and depressive symptoms that some of these adolescents may have experienced have had a tremendous negative impact on their mental health [9]. Moreover, researchers show that the association of normal stress with daily and major life events can exacerbate the aforementioned psychological distresses and increase the risk of depression and anxiety later in life [10].

In 2020, South Korea had the highest age-standardized suicide rate (23.5) among Organisation for Economic Co-operation and Development countries [11], with suicide being the leading cause of death among South Korean individuals aged 10–39 years. While the total number of suicide deaths in South Korea in 2021 showed a slight drop compared with the figures for the preceding year, the suicide rate of individuals aged 10–29 years showed a marked increase [12]. In particular, studies show that sorrow, hopelessness, and depression, all of which can be consequences of the effects of the pandemic, can increase the risk of suicidal ideation [13, 14]. Researchers also demonstrate that measures such as social distancing or lockdowns for preventing COVID-19 infection and mortality are crucial for facilitating the country’s economic and social recovery [15]. Still, it remains that such measures should be accompanied by means of mitigating their effects on mental health.

Accordingly, examinations of adolescent mental health and related problems amid the COVID-19 pandemic could inform the development of specialized education programs that promote positive mental health among this age group. Therefore, this study examined adolescent mental health using data on the health behavior status of South Korean sixth to eighth graders in middle school nationwide during the prolonged COVID-19 pandemic. 

## Materials and methods

### Data and participants

The Korea Youth Risk Behavior Web-based Survey (KYRBS) is an anonymous, self-report online survey that has been conducted annually by the Korea Disease Control and Prevention Agency and the Korean Ministry of Education since 2005. It aims to compute health indices for adolescents and determine their health behaviors. Regarding sampling, the KYRBS is conducted using stratified cluster probability sampling to extract one class from a total of 800 sample schools (i.e., 400 middle schools and 400 high schools) selected by the stratified random extraction method. A total of 57,925 middle and high school seniors were sampled to represent the country’s middle and high school students, which is the target population of this study, by reflecting stratification, cluster, and weight sampling procedures. In this study, data from 54,948 students were used, as some data were excluded as they showed missing values for the variables of interest of this study.

### Variables

Participants were divided into a healthy group, a suicide-ideation group, a suicide-planning group, and a suicide-attempt group based on their responses to suicide-related questions. For the question, “Have you seriously thought about suicide at any time over the past 12 months?”, participants who answered “no” were allocated to the healthy group, while those who answered “yes” were allocated to the suicide-ideation group. Next, those who answered “yes” to the question, “Did you make specific plans to commit suicide at any time over the past 12 months?”, were allocated to the suicide-planning group. Finally, those who answered “yes” to the question, “Have you attempted to commit suicide at any time over the past 12 months?”, were allocated to the suicide-attempt group. Individuals who reported planning and attempting suicide were removed from the suicide-ideation group. Thus, the final suicide-ideation group contained only those individuals who had considered suicide but had not made plans nor attempted to commit suicide. Moreover, the suicide-planning group contained only those who had planned suicide, not those who attempted suicide.

The dependent variables were perceived stress, sadness and despair, loneliness, and generalized anxiety disorder (GAD; Table 1). The basic control variables were sex, education level, academic achievement, father’s highest education level, mother’s highest education level, living arrangement (i.e., whether respondents lived with family members), economic status, impact of COVID-19 on economic status, whether the family received financial assistance, sleep, smoking, drinking, drug use, and smartphone overdependence (i.e., score of 24 or higher on the Smartphone Overdependence Scale, with a range of 10–40, was defined as smartphone overdependence). These variables were selected as they are considered by prior researchers to potentially influence suicidal thoughts and behaviors, and this is further discussed below.

**Table 1:** Definitions of key variables

Variable	Definition
	How much stress do you usually experience?
Stress	1 (“very high”) to 5 (“no stress at all”). Reverse-coded for analysis in this study; thus, a score of 1 represents “no stress at all.” Have you experienced, at any time in the past 12 months, at least two consecutive
Sadness and despair	Weeks of sadness or hopelessness to the point that you put your daily life on hold? 1 (“not in the past 12 months”) or 2 (“yes”)
Loneliness	How frequently over the past 12 months have you felt lonely 1 (“never”) to 5 (“constantly”) Seven-item Generalized Anxiety Disorder scale
Generalized anxiety disorder	(1) Feeling nervous, anxious, or on edge
	(2) Not being able to stop or control worrying
	(3) Worrying too much about different things
	(4) Trouble relaxing
	(5) Being so restless that it is hard to sit still
	(6) Becoming easily annoyed or irritable.
	(7) Feeling afraid, as if something awful might happen
	Each item was scored between 1 (“not bothered at all”) and 4 (“bothered nearly every day”). An overall score was determined by summing the scores for each of the seven items

Sex is considered an important variable in many studies due to related psychological, social, and biological differences. Sex can be associated with suicidal behavior and related risk factors, and men and women tend to express suicidal behavior differently [16]. Education has been shown to be an important influencing factor of social and economic opportunities and resources [17], as low education can be associated with higher social imbalances, negative self-image, mental health problems, among others. Moreover, the lower the education, the more it associates with adaptation problems, emotional pain, and decreased self-esteem at school, which can increase suicidal thoughts or behaviors [18]. Parental education is related to the family environment and socioeconomic status of adolescents [19], in that the lower the level of education of parents, the more they can affect their children’s academic performance, economic stability, and emotional support, and these variables which can associate with suicidal thoughts or behaviors.

The living arrangement of a person can affect the degree of social support and stability in the home [20], and living with the family can act as a protective factor against suicidal thoughts and behaviors. Economic status can increase the risk of suicidal thoughts or behaviors because it can lead to increased psychological stress, anxiety, and lack of social support when people face economic difficulties [18]. Moreover, economic status can be particularly critical when access to social support and resources is limited in unusual situations. Accordingly, in this study, the responses of “upper/middle/middle/lower/lower” to the question “What is the economic status of the family?” were divided into the three groups of low, middle, and high economic status. Whether a family receives financial support relates to the economic stability of the family [14], and while a lack of financial support can negatively affect suicidal thoughts and behaviors, and appropriate financial support can mitigate these risks.

Sleep is essential to physical and mental health [21], and a lack of sleep or sleep disorders can be associated with depression and anxiety, in turn increasing suicidal thoughts or behaviors. In this study, the responses (“very sufficient/sufficient/just not enough/not enough/not at all”) to the question, “Do you think the time you slept in the last seven days is enough for fatigue recovery?”, were categorized into adequate, average, and inadequate sleep. Smoking, drinking, and substance abuse are factors that can negatively affect physical and mental health and influence suicidal thoughts and behaviors [22]. Smartphone addiction and overdependence can negatively affect life, study, and social relationships [23], and smartphone overdependence can be linked to mental health problems and suicidal thoughts and behaviors.

#### Perceived stress

Stress is a major predictor of suicidal ideation in adolescence—a period of strong impulses [24]. The degree of suicidal ideation increases as with the level of stress [25]. The environmental changes provoked by the COVID-19 pandemic represent stressors for adolescents [26]. Still, a study shows that severe stress, sorrow or hopelessness, and suicide-related behaviors declined among South Korean adolescents early in the COVID-19 pandemic [27]. In this study, the question of “How much stress do you experience in your daily life?” was measured using a five-point Likert scale, with the response options being “extremely,” “very much,” “a little,” “rare,” and “none.” According to the online raw data guidelines for adolescent health behavior, responses of “extremely” and “very much” are defined as experiencing stress.

#### Sadness and despair

Depression, an emotional disturbance resulting from a negative perception of oneself, is a major factor in suicidal ideation and suicidal impulse [28]. Among the various symptoms of depression, sadness and despair symptoms are closely related to suicidal ideation among adolescents [29]. Despair is defined as “a state of mind in which there is an entire want of hope” [30, 31]; upon encountering a stressful situation, adolescents can experience despair rather than motivation to solve the problem, and this can result in a higher likelihood of suicidal ideation [32]. In this study, the question used to assess sadness and despair was as follows, “In the past 12 months, have you ever felt sad or hopeless enough to stop engaging in your daily life activities for 2 weeks?” Responses of “I have felt this in the last 12 months” was classified as experiencing sadness and despair.

#### Loneliness

Loneliness is a negative emotion associated with one’s interpersonal relationships, and it can be caused by a lack of attachment to close friends, deficiencies in relationships with significant others, and/or a lack of people with whom to build attachment [33]. Anyone can feel loneliness at least once in their lifetime, but serious cases could lead to mental and physical health problems. The thought of being lonely and alone is strongly associated with suicidal behavior [34, 35]. In this study, loneliness was assessed using the following question, “How often have you felt lonely in the past 12 months?”, which was responded based on the following options: “I didn’t feel lonely at all,” “I rarely felt lonely,” “I often felt lonely,” and “I always felt lonely.” Responses were divided into two groups for the analyses, namely feeling lonely “often” and “”always.”

#### Generalized anxiety disorder

GAD is considered to be independent of depression [36]. GAD encompasses a shorter list of physical symptoms than depression, with an emphasis on worries, concerns, and anxiety. It can be diagnosed only when the individual experiences repeated, multiday episodes of excessive anxiety or worry about various events or activities that persist over a period of six months or longer. Patients with GAD feel they have no control over their worrying, and show significantly higher odds of suicide [37].

In this study, GAD was measured using the Generalized Anxiety Disorder Experience Survey Tool (GAD-7). It comprises 7 items regarding the severity of anxiety experienced during daily life, and a score of 10 is recommended for evaluation of the GAD condition. The optimal sensitivity and validity of the GAD-7 scale have been verified in previous literature, and the details are explained elsewhere [38, 39]. This study changed the categories of previous studies for the responses to this scale, and used two categories, as follows: moderate group (more than 10 points out of 21) and a no anxiety group.

### Statistical analysis

Regarding descriptive statistics, we used frequency and percentage to analyze each study group (i.e., healthy, suicide-ideation, suicide-planning, and suicide-attempt groups). To investigate the statistical differences in categorical variables between groups, chi-square test was used. Next, covariance analysis and post-test analysis were conducted to investigate differences in perceived stress, sadness and despair, loneliness, and GAD. Multiple logistic regression analysis was used to determine the risk for each group regarding these variables, and results were expressed as odds ratios (OR) with a 95% confidence interval (95% CI). All analyses were performed using STATA, version 17.0 (StataCorp) based on multi-layer cluster sampling. The statistical significance level was set at a p < 0.05.

## Results

### Characteristics of the study population

Table 2 shows participants’ general characteristics (N = 57,925) by group: healthy group (n = 48,573; 83.9%), suicide-ideation group (n = 4003; 6.9%), suicide-planning group (n = 1251; 2.2%), and suicide-attempt group (n = 1121; 1.9%).

**Table 2:** Characteristics of the study population (N = 54,948)

Variable		Healthy adolescents (n = 48,573)			Suicide-ideation adolescents (n = 4003)			Suicide-plan adolescents (n = 1251)			Suicide-attempt adolescents (n = 1121)			*χ2*
		No	Weighted no	Weighted %	No	Weighted no	Weighted %	No	Weighted no	Weighted %	No	Weighted no	Weighted %	*p*
Sex	Male	25	1244	53.5	155	75,593	39.6	539	26,365	44.2	382	18,443	34.6	452.84
		880	439		2									
	Female	22	1083	46.5	245	115,455	60.4	712	33,303	55.8	739	34,825	65.4	***
		693	465		1									
	Middle	25	1160	49.9	198	87,276	45.7	690	30,881	51.8	592	26,247	49.3	28.28
Education level		699	962		0									
	High	22	1166	50.1	202	103,772	54.3	561	28,787	48.2	529	27,021	50.7	***
		874	942		3									
	Poor	15	743,845	32.0	159	75,191	39.4	521	24,555	41.2	531	25,215	47.3	239.89
		567			8									
Academic achievement	Middle	14	713,371	30.6	105	50,541	26.5	339	16,152	27.1	271	12,896	24.2	***
		916			9									
	High	18	870,688	37.4	134	65,316	34.2	391	18,961	31.8	319	15,157	28.5	
		090			6									
	Under high school	8853	401,796	17.3	786	35,023	18.3	225	10,241	17.2	213	9738	18.3	9.77
Fathers’ education level		20	997,547	42.9	168	83,770	43.8	509	25,588	42.9	421	21,623	40.6	
		023			5									
	Unknown	19	928,561	39.9	153	72,255	37.8	517	23,839	40.0	487	21,908	41.1	
		697			2									
	Under high school	10	46,541	2.0	962	44,336	23.2	266	12,279	20.6	262	12,792	24.0	33.88
Mothers’ education level		166												
	College or higher	19	976,940	42.0	160	78,578	41.1	481	23,833	39.9	423	0.549	38.6	***
		695			8									
	Unknown	18	881,423	37.9	143	68,134	35.7	504	23,556	39.5	436	19,927	37.4	
		712			3									
	With family	46	2244	96.4	377	181951	95.2	117	56,445	94.6	1	48,463	91.0	108.63
		379	820		2			0			011			
Living arrangement	Without Family	2194	83,084	3.6	231	9097	4.8	81	3223	5.4	110	4805	9.0	***
Economic Status	Low	5810	268,411	11.5	837	38,224	20.0	273	12,013	20.1	292	13,077	24.5	464.42
	Middle	23	1116	48.0	181	86389	45.2	556	26,273	44.0	447	21,166	39.7	***
		582	796		2									
	High	19	942,697	40.5	135	66436	34.8	422	21,383	35.8	382	19,025	35.7	
		181			4									
	Not at all	14	718,326	30.9	940	46,767	24.5	305	14,820	24.8	256	12,535	23.5	479.25
Economic status changed owing to COVID-19		767												
	Really	19	939,020	40.3	153	73,861	38.7	430	20,294	34.0	357	16,852	31.6	***
		518			6									
	Changed	11	549,901	23.6	116	53,584	28.0	389	18,160	30.4	333	16,050	30.1	
		698			3									
	Critically	2590	120,658	5.2	364	16,837	8.8	127	6394	10.7	175	7831	14.7	
Financial assistance	No	43	2121	91.1	343	165,410	86.6	105	50,486	84.6	934	45,084	84.6	187.87
		961	168		7			3						
	Yes	4612	206,736	8.9	566	25,638	13.4	198	9182	15.4	187	8184	15.4	***
	Adequate average	15	745,642	32.0	671	31,280	16.4	244	11,509	19.3	188	9115	17.1	1046.1
		721												4
Sleep		16	795,703	34.2	126	60,185	31.5	344	16,039	26.9	302	14,316	26.9	***
		750			0									
	Inadequa te	16	786,559	33.8	207	99,582	52.1	663	32,121	53.8	631	29,837	56.0	
		102			2									
	No	43	2096	90.0	330	158675	83.1	102	48,923	82.0	769	36,822	69.1	705.07
Smoking		766	121		8			4						
	Yes	4807	231,783	10.0	695	32,373	16.9	227	10,745	18.0	352	16,447	30.9	***
	No	33	1593	68.4	214	102,850	53.8	687	32,578	54.6	475	23,150	43.5	713.53
		285	087		4									
Alcohol use														
	Yes	15	734,817	31.6	185	88,198	46.2	564	27,090	45.4	646	30,118	56.5	***
		288			9									
	No	48	2316	99.5	393	187,475	98.1	122	58,300	97.7	104	49,752	93.4	662.34
Drug use		346	576		1			6			0			
	Yes	227	11,328	0.5	72	3573	1.9	25	1368	2.3	81	3516	6.6	***
Smartphone overdependence	No	37	1776	76.3	243	114,313	59.8	809	37,702	63.2	721	33,990	63.8	685.41
		209	021		4									
	Yes	11	551883	23.7	156	76,735	40.2	442	21,966	36.8	400	19,278	36.2	***

^*^p < .05, **p < .01, ***p < .001

The healthy group had more boys (53.5%) than girls, had a similar ratio of high school students (49.9%) to middle school students (50.1%), and featured the highest percentage of students with high academic achievement (37.4%). Many of the students in this group had parents with college or higher education levels (fathers = 42.9%, mothers = 42.0%). Moreover, 96.4% lived with family members, while 48.0% were middle class. Next, 5.2% stated that they had experienced low economic status as a result of the COVID-19 pandemic, while 91.1% stated that their families had not received any financial assistance. Finally, 34.2% reported inadequate sleep, 10.0% were smokers, 31.6% were alcohol users, 0.5% had experience using drugs, and 23.7% had smartphone overdependence.

The suicide-ideation group comprised mostly girls (60.4%), a higher percentage of high school students (54.3%) than middle school students, and students with poor academic achievement (39.4%). Many of the students had parents with college or higher education levels. Moreover, 95.2% lived with family members, while 45.2% were middle class. Next, 8.8% stated that they had experienced low economic status as a result of the COVID-19 pandemic, while 86.6% stated that their families had not received any financial assistance. Finally, 52.1% reported inadequate sleep, 16.9% were smokers, 46.2% were alcohol users, 1.9% had experience using drugs, and 40.2% had smartphone overdependence.

The suicide-planning group comprised mostly girls (55.8%), a higher percentage of middle school students (51.8%) than high school students, and students with poor academic achievement (41.2%). Many of the students had parents with college or higher education levels. Moreover, 94.6% lived with family members, while 44.0% were middle class. Next, 10.7% stated that they had experienced low economic status as a result of the COVID-19 pandemic, while 84.6% stated that their families had not received any financial assistance. Finally, 53.8% reported inadequate sleep, 18.0% were smokers, 45.4% were alcohol users, 2.3% had experience using drugs, and 36.8% had smartphone overdependence.

The suicide-attempt group consisted predominantly of girls (65.4%), had a similar ratio of high school (49.3%) and middle school students (50.7%), and was the group with the highest percentage of students with poor academic achievement (47.3%). About 40.6% of the fathers of students in this group were college graduates, and 41.1% reported not knowing the father’s education level. The mothers of most students reportedly had college or higher education. Moreover, 91.0% lived with family members, while 39.7% were middle class. Next, 14.7% stated that they had experienced low economic status as a result of the COVID-19 pandemic, while 84.6% stated that their families had not received any financial assistance. Finally, 56.0% reported inadequate sleep, 30.9% were smokers, 56.5% were alcohol users, 6.6% had experience using drugs, and 36.2% had smartphone overdependence.

## Discussion

Since the outbreak of COVID-19 in Hubei, China, in December 2019, restrictions have been imposed on people’s daily lives to address the threat of the virus; however, these restrictions have had prolonged negative consequences. The severing of social relationships owing to measures such as social distancing has resulted in mental health problems in the general population, and fear of quarantine has led to depression, anxiety, stress, and despair, thereby impairing people’s quality of life. In particular, 59.8% of adolescents in South Korea have reported dominant feelings of “anxiety and worry” as a result of the COVID-19 pandemic [37]. Thus, this study analyzed the factors associated with mental health issues and suicide among adolescents by categorizing a representative sample of South Korean adolescents into four groups: a healthy group, suicide-ideation group, suicide-planning group, and suicide-attempt group. We sought to specifically identify the predictors of related mental health issues. The following results were obtained.

First, among 57,925 South Korean adolescents, 6.9% showed suicidal ideation, 2.2% reported planning suicide, and 1.9% reported attempting suicide over the 12 months preceding the data collection. These rates are lower than those reported in a 2019 study conducted in the United States among children and adolescents, wherein 18.8% reported seriously considering attempting suicide, 15.7% reported planning suicide, and 8.9% reported attempting suicide [41]. The above data show that adolescents in South Korea and the US are more likely to engage in suicidal ideation than to actually plan or attempt suicide [42]. However, in Ghana’s Global School-based Student Health Survey conducted in 2012 for high school students, the prevalence of suicidal thoughts, suicide plans, and suicide attempts was 18.2%, 22.5%, and 22.2%, respectively. Therefore, further research is needed on these differences in suicide-related variables between countries [43]. Moreover, although there is a large discrepancy between suicidal ideation and suicidal behavior among adolescents, this contrasts the situation of middle-aged and older adults [44]. Still, as adolescents have relatively less life experience and tolerance of various forms of shocks and stressors than do their older counterparts, they can be more impulsive regarding suicide. This indicates the need for national policies in South Korea that exclusively target suicide prevention among adolescents.

Second, all demographic characteristics examined in this study, except for fathers’ highest education level, significantly predicted adolescents’ mental health status. In the healthy group, male high schoolers with higher academic achievement were mentally healthier. In the suicide-ideation and suicide-attempt groups, female high schoolers with poor grades showed a higher risk of mental health problems. When compared to the healthy group, the risk of mental health problems was higher among students who were not living with family members, those who had a lower socioeconomic status, and those who reported planning or attempting suicide. The risk of stress, sadness and despair, loneliness, or GAD was higher among those who had experienced greater financial difficulty as a result of the COVID-19 pandemic. The risk was also higher among those with inadequate sleep, smokers, alcohol users, those with experience using drugs, and those with smartphone overdependence as compared to their counterparts. The level of financial assistance received had little effect.

The risk to attempt suicide was higher among female high schoolers with “poor” academic achievement, those living with non-family members, and those with lower economic status as compared to their counterparts. The risk of suicidal ideation, suicide planning, and suicide attempt was higher among those with inadequate sleep, smokers, alcohol users, those with experience using drugs, and those with smartphone overdependence. The findings for sex in this study are similar to those of previous South Korea-based studies, in which the risks of suicidal ideation, suicide planning, and suicide attempt were higher among female than male students [45–47]. A possible reason for this is that male students tend to interpret an event cognitively, while female students are more heavily influenced by and sensitive to the emotional aspects of the event [47, 48]. Between 2010 and 2017, the mean suicide rate among adolescents aged 10–19 years in South Korea was 5.45% for boys and 4.30% for girls, differing from the US rates of 7.57% for boys and 2.57% for girls [49]. Both the South Korean and US data show differences between sexes, which indicates that a sex-specific approach should be taken to address this issue.

As aforementioned, the results showed that sleep, smoking, alcohol, drugs, and smartphone overdependence were associated with the risk of suicide. This shows the necessity of helping students recover psychologically and improve their quality of life; this can be done by providing various forms of support for their mental and psychological conditions within the educational system, as past studies also show that drinking, smoking, drug use, or excessive experience of smartphones in adolescence harms physical health [22, 23, 50]. According to the SEYLE Cluster Randomized Controlled Trial (German Clinical Trials Register DRKS00000214), which encompassed 11,110 high school students in 10 EU countries, specific school-based suicide prevention interventions were effective in mitigating the dangerous effects of self-harm behavior; these interventions could also be further developed by increasing parental participation [51]. Parental support and encouragement may be one of the key factors for adolescents to be able to avoid suicidal thoughts. This suggests that the role of parents in recognizing adolescents as part of the family is essential for adolescent suicide prevention, as parental affirmation may help adolescents perceive themselves as valuable. Thus, to promote adolescent suicide prevention in South Korea, education systems and parents should facilitate or provide various forms of psychological support to adolescents, as these efforts may help strengthen adolescent mental health and, in turn, quality of life.

Furthermore, humanity has now already experienced how much our daily lives can change very quickly upon the onset of a pandemic, such as that provoked by COVID-19. Thus, we should conduct in-depth discussions on how our daily lives would be like in the upcoming post-COVID-19 period. To systematically respond to these forthcoming periods of human history, we could develop and apply digital platform education programs aimed at improving adolescent health behaviors and mental health, especially among those who had their lives impacted by the COVID-19 pandemic. Once more, education systems can play a significant role on the topic, as they can provide adolescents with various forms of psychological support so as to strengthen their mental health and quality of life. 

Third, we comparatively analyzed mental health and suicide-related factors. The results showed that negative emotions such as depression among adolescents increase the odds of their progressing from suicidal ideation to attempting suicide. This supports previous findings that adolescents with emotional problems exhibit high levels of depressive symptoms, stress, and impulse to drop out of school [52], and that loneliness is a powerful predictor of suicidal ideation [53, 54]. Contrastingly, Naragon-Gainey and Watson [55] reported that while depression is a significant factor in suicidal ideation, GAD is not. Another study shows that emotional intelligence associates with health indices, and that decreasing emotional intelligence relates to increasing suicidal behavior [56]. In an analysis of the components and behavioral risk reduction of adolescent mental health programs for those aged 10–19 across 18 studies, the interventions were shown to universally improve adolescent mental health and reduce risk behaviors [57]. Thus, programs that cultivate one’s ability to control and positively manage emotions should be provided for adolescents who are psychologically unstable and who are repeatedly exposed to negative emotions; this could help to promote psychological stability and functionality.

Fourth, we identified factors that predict adolescents’ mental health status. Specifically, sex, academic achievement, economic status, impact of COVID-19 on economic status, sleep, alcohol use, drug use, smartphone overdependence, and suicide-related factors were identified as significant predictors. Many studies have similarly reported that sex, academic achievement, economic status, health behaviors, and emotional characteristics influence adolescents’ mental health and suicidal ideation [58, 59]. Further, studies exploring the influence that COVID-19-induced changes in subjective economic status have on adolescents’ stress have found that deterioration in economic status has a negative impact on adolescents’ mental health [60–62].

Past studies on suicide have generally examined predictors of suicidal ideation. Contrastingly, this study classified adolescents into four groups, namely the healthy, suicide-ideation, suicide-planning, and suicide-attempt groups. Thus, the significance of this study lies in considering suicide not as a simple, isolated event, but rather as a complex and dynamic process that forms a continuum from suicidal ideation through planning suicide to attempting suicide. Thus, in examining the demographic characteristics and the transition to suicidal attempt in individuals in the healthy group, we shed light on the specific directions that can be taken for interventions targeting each stage of mental health crisis among adolescents, from suicidal ideation to suicidal planning and suicide attempt. Further, while past studies have generally focused on depression when examining factors associated with suicide in adolescents, we confirmed that sadness and despair and GAD elevate the risk of suicidal ideation, suicide planning, and attempting suicide. These findings collectively showcase the need for programs that, through accurate mental health evaluations, focus on these factors and enable the early screening and implementation of interventions for adolescents at risk of suicide. Another strength of this study is that it provides an opportunity to identify new means of maintaining adolescents’ mental health amid the prolonged COVID-19 pandemic.

### Comparisons of stress, sadness and despair, loneliness, and GAD among the different groups of adolescents

**Fig. 1:**
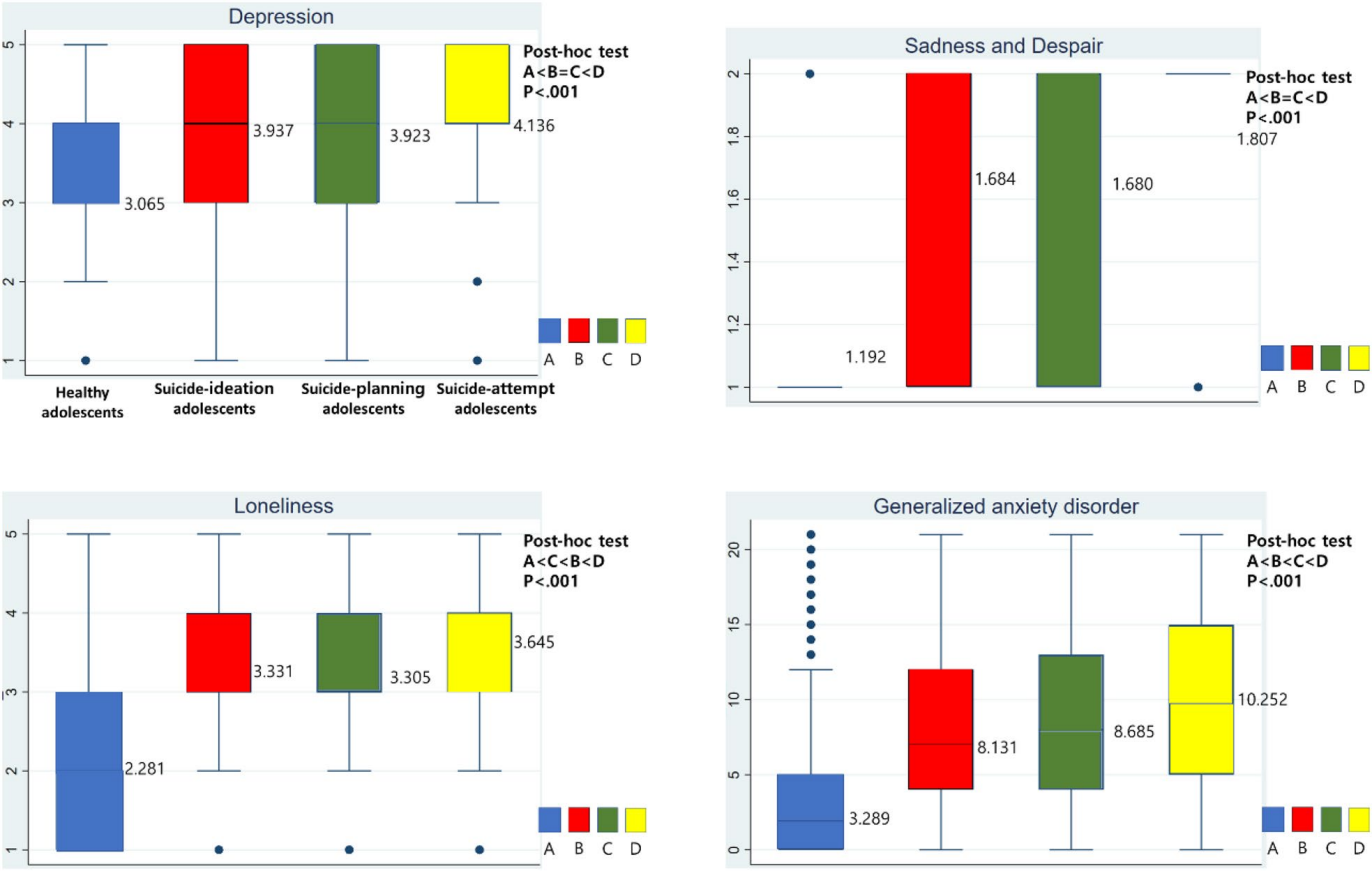
Analysis of covariance and post-hoc test for stress, sadness and despair, loneliness, and generalized anxiety disorder in the health, suicide-ideation, suicide-planning, and suicide-attempt groups. The basic control variables included sex, education level, academic achievement, father’s highest education level, mother’s highest education level, living arrangement, economic status, impact of COVID-19 on economic status, whether the family received financial assistance, sleep, smoking, drinking, drug use, and smartphone overdependence

Comparisons of the key variables are displayed in Fig. 1. The suicide-attempt group showed the highest level of stress (4.136); this group was followed by the suicide-ideation group (3.938), suicide-planning group (3.923), and healthy group (3.065). The difference was significant (p < 0.001).

The suicide-attempt group showed the highest level of sadness and despair (1.807); this group was followed by the suicide-ideation group (1.684), suicide-planning group (1.680), and healthy group (1.192). The difference was significant (p < 0.001).

The suicide-attempt group showed the highest level of loneliness (3.645); this group was followed by the suicide-ideation group (3.331), suicide-planning group (3.305), and healthy group (2.281). The difference was significant (p < 0.001).

The suicide-attempt group showed the highest level of GAD (10.252); this group was followed by the suicide-planning group (8.685), suicide-ideation group (8.131), and healthy group (3.289). The difference was significant (p < 0.001).

#### Predictors of mental health status among South Korean adolescents

Table 3 shows the results of the multiple logistic regression analysis that was performed to identify the predictors of mental health status among South Korean adolescents.

**Table 3:** Multiple logistic regression analysis on stress, sadness and despair, loneliness, and GAD (N = 54,948)

Variable		Stress		Sadness and despair		Loneliness			GAD		
		ORs 95% pCI)		ORs (95% P CI)		ORs (95% P CI)			ORs (95% P CI)		
		1.779	***	1.632	***	1.76		***	1.581		***
						8					
Sex (Ref. = Male)	Female										
		1.700	1.862	1.561	1.707	1.70		1.836	1.517		1.646
						3					
		1.113	***	0.999		1.00			1.071		**
Education level (Ref. = Middle)	High					2					
		1.063	1.166	0.955	1.046	0.96		1.041	1.027		1.116
						4					
Academic achievement	Middle	0.989		0.843	***	0.89		***	0.908		***
						3					
(Ref. = Poor)		0.935	1.046	0.799	0.890	0.85		0.936	0.863		0.954
						3					
		0.901	***	0.770	***	0.94		**	0.937		*
						0					
	High										
		0.852	0.953	0.729	0.813	0.89		0.984	0.892		0.985
						7					
	College or higher	1.134	**	1.086	*	1.07		*	1.060		
						3					
Fathers’ education		1.055	1.218	1.013	1.164	1.01		1.139	0.995		1.130
						1					
Level (Ref. = Below high school)											
		1.094	*	1.037		0.96			1.005		
	Unknown					1					
		1.005	1.191	0.956	1.125	0.89		1.031	0.932		1.083
						6					
Mothers’ education	College or higher	0.975		1.034		1.00			1.019		
						2					
level (Ref. = Below high		0.910	1.045	0.968	1.105	0.94		1.061	0.959		1.083
school)						7					
		0.979		1.010		0.95			0.928		*
	Unknown					3					
		0.900	1.065	0.933	1.094	0.88		1.020	0.862		0.999
						9					
		0.976		1.012		1.18		***	0.986		
Living arrangement (Ref. = With family)	Without family					2					
		0.877	1.086	0.916	1.117	1.08		1.290	0.899		1.082
						4					
		0.781	***	0.857	***	0.75		***	0.767		***
						4					
	Middle										
Economic status (Ref. = Low)		0.721	0.847	0.801	0.917	0.70		0.801	0.721		0.817
						9					
	High	0.704	***	0.937		0.71		***	0.743		***
						5					
		0.647	0.766	0.870	1.009	0.66		0.764	0.693		0.795
						9					
		1.310	***	1.068	*	1.33		***	1.196		***
	Really					6					
		1.245	1.379	1.011	1.128	1.27		1.398	1.137		1.257
						7					
Economic status changed owing to COVID-19 (Ref. = Not at all)		1.507	***	1.294	***	1.58		***	1.382		***
	Changed					5					
		1.418	1.603	1.218	1.376	1.50		1.669	1.307		1.462
						5					
		1.540	***	1.645	***	1.53		***	1.500		***
						6					
	Critically										
		1.382	1.715	1.497	1.808	1.40		1.674	1.370		1.641
						9					
Financial assistance (Ref. = No)	Yes	1.005		1.147	***	1.13		***	1.115		**
						3					
		0.927	1.089	1.068	1.233	1.06		1.208	1.042		1.193
						2					
		2.242	***	1.399	***	1.66		***	1.639		***
	Average					6					
		2.133	2.358	1.320	1.483	1.59		1.743	1.555		1.728
						2					
Sleep (Ref. = Adequate)											
		3.197	***	2.132	***	2.20		***	2.759		***
						6					
	Inadequate										
		3.024	3.381	2.016	2.255	2.10		2.311	2.621		2.905
						7					
		0.945		1.405	***	1.25		***	1.029		
						6					
Smoking (Ref. = No)	Yes										
		0.872	1.024	1.310	1.507	1.17		1.340	0.961		1.101
						6					
Drinking (Ref. = No)	Yes	1.148	***	1.396	***	1.37		***	1.166		***
						3					
		1.089	1.210	1.329	1.467	1.31	1.433		1.114	1.221	
						5					
		1.510	*	2.216	***	1.39	**		2.056	***	
						7					
Drug use (Ref. = No)	Yes										
		1.072	2.127	1.742	2.820	1.09	1.781		1.621	2.608	
						6					
		1.724	***	1.484	***	1.81	***		2.436	***	
Smartphone overdependence (Ref. = No)	Yes					7 1.74					
		1.627	1.828	1.415	1.556	1	1.897		2.333	2.545	
		6.233	***	7.080	***	4.95	***		5.227	***	
						0					
	Ideation										
Suicide (Ref. = Healthy)		5.237	7.417	6.581	7.616	4.52	5.411		4.844	5.640	
						8					
	Plan	3.211	***	7.144	***	3.59	***		5.293	***	
						5					
	Attempt	2.563	4.022	6.300	8.101	312	4.142		4.641	6.037	
						1					
		6.003	***	11.911	***	6.13	***		6.602	***	
						6					
						5.09					
							7.384		5.678	7.676	
						8					

*GAD* generalized anxiety disorder

*p < .05, **p < .01, ***p < .001

The ORs for stress, sadness and despair, loneliness, and GAD were higher among boys than girls (stress OR = 1.779; sadness and despair OR = 1.632; loneliness OR = 1.768; GAD OR = 1.851).

Regarding education level, the ORs for stress (OR = 1.113) and GAD (OR = 1.071) were higher among high schoolers than middle schoolers. Regarding academic achievement, the ORs were lower among students with good grades than those with poor grades (stress in high schoolers OR = 0.901; sadness and despair in middle schoolers OR = 0.843, in high schoolers OR = 0.770; loneliness in middle schoolers OR = 0.893, in high schoolers OR = 0.940; GAD in middle schoolers OR = 0.908, in high schoolers OR = 0.937).

The ORs for stress, sadness and despair, and loneliness were higher among students whose fathers were college graduates or higher than among those whose fathers had high school or lower education (stress in college or higher OR = 1.134, in unknown OR = 1.094; sadness and despair in college or higher OR = 1.086; loneliness in college or higher OR = 1.073). The OR for GAD was higher among students whose mothers were college graduates than among those whose mothers had below high school education (OR = 1.083).

Regarding living arrangement, the OR for loneliness (OR = 1.182) was higher among students who lived with a non-family member than those who lived with a family member.

Regarding economic status, the ORs were lower among students with middle and high status than those with low status (stress in middle OR = 0.781, in high OR = 0.704; sadness and despair in middle OR = 0.857; loneliness in middle OR = 0.754, in high OR = 0.715; GAD in middle OR = 0.767, in high OR = 0.743).

The ORs were higher among those who had experienced low economic status as a result of the COVID-19 pandemic than among those who did not experience this (stress in really OR = 1.310, in changed OR = 1.507, in critically OR = 1.540; sadness and despair in really OR = 1.068, in changed OR = 1.294, in critically OR = 1.645; loneliness in really OR = 1.336, in changed OR = 1.585, in critically OR = 1.536; GAD in really OR = 1.196, in changed OR = 1.382, in critically OR = 1.500).

Regarding financial assistance, the ORs for sadness and despair, loneliness, and GAD were higher among students who lived with a non-family member than those who lived with a family member (sadness and despair OR = 1.147; loneliness OR = 1.133; GAD OR = 1.115).

The ORs were higher among those who had adequate and average sleep than among those who had inadequate sleep (stress in average OR = 2.242, in inadequate OR = 3.197; sadness and despair in average OR = 1.399, in inadequate OR = 2.132; loneliness in average OR = 1.666, in inadequate OR = 2.206; GAD in average OR = 1.639, in inadequate OR = 2.759).

The ORs for sadness and despair (OR = 1.405) and loneliness (OR = 1.256) were higher among smokers than non-smokers. The ORs for all four mental health conditions were higher among those with inadequate sleep, alcohol users, those with drug user experience, and individuals with smartphone overdependence.

## Conclusions

Suicide is an outcome of complicated, dynamic, and unique interactions among numerous contributors [60]. Thus, educational programs and campaigns that raise awareness of behaviors that increase the risk of suicide (e.g., smoking and drug and alcohol use) should be frequently conducted to improve adolescents’ mental health status. Additionally, individualized mental health programs should be developed to help adolescents manage their mental health, as this would contribute to addressing the emotional aspects and other factors associated with suicide (e.g., stress, depression, loneliness). Childhood and adolescence are critical periods for physical and emotional growth and development, and COVID-19-induced changes in daily life can have a tremendous impact on young people’s future lives. Hence, as the COVID-19 pandemic continues, it is important to pay attention, both in the social context and in research, to restrictions in daily life that can affect adolescents’ mental health, and to mitigate these effects where possible.

### Limitations

First, we only analyzed the variables present in the 16th KYRBS data; thus, the findings cannot be generalized to the entire adolescent population in South Korea or populations in other countries. Still, measures such as social distancing and outdoor activity restrictions to prevent the spread of COVID-19 are likely to have affected adolescents in other countries. Therefore, future research on how the lives of adolescents have changed after COVID-19 across different countries can continue to promote our understanding of this phenomenon in different cultural contexts, as well as enable for comparisons between countries. Specifically, these future studies can compare their data from different cultural contexts with those of this study on South Korean adolescents, as this may yield more awareness of the commonalities and differences of the impact of COVID-19—and the response measures to the pandemic—on the lives and mental health of adolescents. These efforts will contribute to the welfare and development of adolescents. Moreover, despite this limitation, it is highly likely that the effects of measures for COVID-19 prevention, such as social distancing and outdoor activity restrictions, were also felt by adolescents in countries other than South Korea.

Second, as suicide occurs during moments of heightened cognitive and emotional vulnerability, both cognitive and emotional aspects should be examined to gain an in-depth understanding of (and identify means of disrupting) the process preceding a suicide attempt. Therefore, to understand the thinking patterns and processes before suicide attempts from a cognitive perspective, as well as develop cognitive interventions or self-treatment methods to prevent suicide attempts, it is necessary to investigate and analyze negative thoughts, self-evaluation, and despair before such attempts. Developing methods to detect and control changes in emotional states may allow for stakeholders to take measures to prevent and alleviate emotional vulnerability by using emotional control technology, strengthening interpersonal and social support, and emotional expression methods. Third, continuous research on adolescent mental health—as opposed to single-year surveys—is required to prepare for challenges associated with potential future pandemics.

## Data Availability

The data are available from the KCDA website (https://www.kdca.go.kr/yhs/).

## Abbreviations

COVID-19: Coronavirus disease 2019
GAD: Generalized anxiety disorder
KYRBS: Korea Youth Risk Behavior Web-based Survey
OR: Odds ratio
SARS: Severe acute respiratory syndrome
